# Regulation of the Human Papillomavirus Lifecyle through Post-Translational Modifications of the Viral E2 Protein

**DOI:** 10.3390/pathogens10070793

**Published:** 2021-06-23

**Authors:** Leny Jose, Timra Gilson, Elliot J. Androphy, Marsha DeSmet

**Affiliations:** 1 Department of Dermatology, Indiana University School of Medicine, Indianapolis, IN 46202, USA; lenyjose@iu.edu (L.J.); tgilson@iupui.edu (T.G.); eandro@iu.edu (E.J.A.); 2 Department of Microbiology and Immunology, Indiana University School of Medicine, Indianapolis, IN 46202, USA

**Keywords:** HPV E2, replication, PTM

## Abstract

The human papillomavirus (HPV) is a DNA tumor virus that infects cutaneous and mucosal epithelia where high-risk (HR) HPV infections lead to cervical, oropharyngeal, and anogenital cancers. Worldwide, nearly 5% of all cancers are caused by HR HPV. The viral E2 protein is essential for episomal replication throughout the viral lifecycle. The E2 protein is regulated by phosphorylation, acetylation, sumoylation, and ubiquitination. In this mini-review, we summarize the recent advancements made to identify post translational modifications within E2 and their ability to control viral replication.

## 1. Introduction

Human papillomaviruses (HPV) are double stranded covalently closed circular DNA viruses that infect cutaneous and mucosal epithelia. These viruses are the causative agents of cervical, anal, and oropharyngeal carcinomas. After infection of the basal epithelium, the viral genome localizes to the host nucleus and replicates to a low copy number as an autonomous plasmid or episome. This tightly controlled process is mediated by the viral proteins E1 and E2 [1]. Papillomavirus (PV) genomes duplicate and partition to progeny cells during mitosis in the basal and suprabasal cells [2,3]. The viral replicative lifecycle is linked to the differentiation status of the infected epithelium [4,5]. As infected cells migrate into the upper epithelial strata where differentiation occurs, viral episomes amplify to hundreds of copies.

The E2 protein is necessary for replication of the viral genome, transcriptional activation of the viral early promoter, and tethering of viral genomes to host mitotic chromosomes in basal cells during cell division. The E2 protein is composed of about 200 amino acid transactivation domains (TAD) followed by a non-conserved non-structured region. The E2 TAD mediates interactions with cellular and viral proteins to regulate transcription and replication. At the C-terminus is an approximately 85–120 amino acid DNA binding domain (DBD) that folds as a dimeric beta barrel. The E2 DBD binds with high affinity to inverted palindrome ACCN_6_GGT DNA sequences in the PV genome including at the origin of replication (ori) [6]. The E1 protein is a DNA helicase and is recruited to the ori by E2. All the PV E2 proteins share this similar protein structure and functionalities. The activities and cellular binding partners of E2 have been reviewed [6,7].

The biological activity of E2 is diverse and its function is dependent on not only the levels of E2 during the different stages of the viral replicative lifecycle but also by post-translational modifications (PTMs) within E2. Interestingly, many of these PTMs are essential for modulating E2-host interactions. Advances in mass spectrometry techniques and development of PTM specific antibodies have made it possible to discover a series of E2 regulatory PTMs. In this mini review, we discuss recent advances in the host regulation of the E2 protein mediated by PTMs such as phosphorylation, acetylation, sumoylation, and ubiquitination and highlight their roles in the modulating the viral lifecycle.

## 2. Serine/Threonine Phosphorylation

Protein phosphorylation is a reversible post-translational modification, often regulatory in nature, and is fundamental to cell signaling mechanisms. The HPV-8, HPV-11, HPV-16, HPV-31, bovine papillomavirus (BPV-1) and cottontail rabbit papillomavirus (CRPV) E2 proteins are phosphoproteins [8,9,10,11,12,13,14,15]. The first phosphorylations within E2 were identified on serine 298 and 301 residues, located in the non-conserved hinge region of BPV-1 E2, by peptide mapping and site-directed mutagenesis [16]. These serine residues are part of a canonical PEST (proline, glutamic acid, serine, and threonine) sequence, which regulates protein degradation. Casein kinase 2 (CK2) phosphorylates S298 and S301 to target the E2 protein for degradation [17,18]. Mutation of serine to alanine (S301A) increased the half-life by preventing E2 ubiquitination [19] and increased BPV-1 genome copy numbers [20].

Serine phosphorylations at amino acids 235, 240, 277 within the hinge domain of BPV-1 E2 were identified using mass spectrometry [21]. However, S235, when mutated to alanine had an impaired oncogenic transformation phenotype in mouse C127 cells only when S290, S298, and S301 were also mutated to alanine [21]. The authors found that serine phosphorylation at these residues is important for viral genome retention. The location of these phospho-dependent regulations within the hinge region of E2 are unique to BPV-1.

Similar to BPV-1, many of the serine/threonine phosphorylations identified with HPV E2 proteins have been located in the non-conserved hinge region. Johansson et al. found that HPV-16 E2 stability, DNA binding affinity, and phosphorylation were highest during S phase in U2OS cells [10]. Upon further investigation, the cell cycle regulator cyclin dependent kinase 2 (Cdk2) was found to phosphorylate HPV-16 E2 in vitro. Sequence analysis revealed a Cdk2 consensus sequence (TP) within HPV-16 E2 at amino acids 286–287. Mutation of threonine 286 to alanine reduced Cdk2-mediated phosphorylation within E2 but did not alter protein stability during S-phase [10]. Thus, the functional consequences of phosphorylation at T286 within E2 remain unclear.

Serine 243 in the HPV-16 E2 hinge region is an important residue for E2 association with mitotic chromosomes [9] and was identified as a phosphorylation site by mass spectrometry along with S207. Serine 243 is part of the consensus RXXS domain. Mutation of serine 243 to the phosphomimetic glutamic acid (E) or aspartic acid (D) had no effect on E2 binding to metaphase/anaphase chromosomes. However, preventing phosphorylation through mutation to alanine, asparagine (N), or glutamine (Q) decreased the half-life of E2 and prevented colocalization with the Bromodomain-containing protein 4 (Brd4) at mitotic chromosomes. Similar mutagenesis of S207 did not result in altered Brd4 binding [9]. The association of some HPV types E2 with Brd4 is involved in genome segregation during cellular division and is mediated by the interaction between the TAD of E2 and the C-terminal domain (CTD) of Brd4 [15,22,23]. This study suggests that E2 phosphorylation regulates mitotic segregation during the maintenance stage of the viral lifecycle. Similar to BPV E2, the importance of phosphorylation in the non-conserved hinge region among other HPVs has not be determined.

Most recently, HPV16 E2 was found to be phosphorylated at S23 and confirmed by employing specific phosphoantibodies [24]. This residue resides in the alpha-helix of the TAD. Interestingly, this phosphorylation, which is also mediated by CK2, promoted the interaction of E2 with topoisomerase binding protein 1, TopBP1, and was predicted to modulate partitioning of the viral genomes during mitosis [24]. Unpublished mass spectrometry data from our lab also identified S23 phosphorylation in BPV-1 E2, which further emphasizes the role of conserved phosphorylation sites across PVs.

Protein kinase A phosphorylates HPV-8 E2 within the hinge region at serine 253, which is analogous to S243 in HPV-16 E2 [15]. Phosphorylation of this RXXS motif occurs during S-phase to stabilize the protein. The RXXS motif at amino acids 250–253 is conserved in beta-PV E2 proteins [6]. Serine 253 is necessary for binding E2 to chromatin during mitosis [15,22]. It is likely that S253 phosphorylation of E2 proteins of cutaneous beta PVs regulates E2 binding and partitioning of genomes during cellular division.

HPV-1 and HPV-5 hinge regions contain multiple SR/RS motifs, such as those phosphorylated by the Serine-Arginine specific protein kinase 1 (SRPK1). Overexpression of SRPK1 causes a gel mobility shift in HPV-5 E2, suggesting at least one of the 27 hinge SR motifs within E2 could be a kinase target. Phosphorylation changes localization of nuclear HPV-5 E2 to the cytoplasm [25]. However, SRPK-1 phosphorylation of HPV-1 E2 targets the protein to the nucleolus [26]. Of the four hinge region SR motifs in HPV-1 E2, S265 and S267 were confirmed as the major kinase targets, with possibly S281 as a minor target [27].

The E8^E2 protein is composed of a short peptide (~12 aa) of the E8 reading frame fused to the carboxy-terminal E2 DBD. The HPV-31 E8^E2 spliced viral transcript (1296^3295) when over-expressed in cell culture models functions as a repressor for PV replication; however, the native protein has not been detected [28]. Van de Poel et al. identified phosphorylation at serine residues 78, 81, and 100 within HPV-31 E8^E2 by mass spectrometry [29]. These phospho-serine residues map to serine 268, 269, and 288 in full length HPV-31 E2. S78 was the only phosphorylation site detected using a phos-tag system in keratinocytes. Phosphorylation at this serine is required for transcriptional repression of cellular genes by E8^E2 but not for HPV replication [29].

Protein dephosphorylation is catalyzed by protein phosphatases. Only one serine/threonine phosphatase has been shown to interact with and dephosphorylate HPV-16 E2. The HPV-16 E2 TAD binds to the nuclear receptor interaction protein (NRIP) to recruit the calcium and calmodulin dependent serine/threonine phosphatase calcineurin. Dephosphorylation of HPV-16 E2 by calcineurin increases E2 stability but the effect on the viral replicative lifecycle remains unknown [26].

## 3. Tyrosine Phosphorylation

Less than 1% of all protein phosphorylations are on tyrosine residues [30]. During tyrosine phosphorylation, the phosphate is located further away from the peptide backbone and, therefore, has a unique binding specificity compared to serine/threonine phosphorylation and contains more specific binding pockets for protein docking [31]. Evolution of phospho-tyrosine specific antibodies and improved phospho-tyrosine peptide detection by mass spectrometry have made it possible to discover a number of tyrosine phosphorylations previously unidentified.

E2 tyrosine phosphorylation was first identified in BPV-1 E2 at residue 102 in the TAD using mass spectrometry [32]. Tyrosine 102 is conserved among 28 HPVs including HR HPV-16 and HPV-31 but not in HR HPV-18. When BPV-1 E2 Y102 was mutated to glutamate to impart a negative charge similar to phosphotyrosine, this mutant was unable to stimulate transient and stable replication and was transcriptionally impaired. BPV-1 E2 Y102E was defective for binding to E1 and to the C-terminal motif (CTM) of the chromatin modulator Brd4 [32]. Initially, this was surprising since in the co-crystal structure, a twenty amino acid Brd4 C-terminal peptide forms a helix that binds across the first three α-helices on the outer surface of the BPV E2 TAD [33,34]. The CTM region is the p-TEFb binding region of Brd4 (aa 1209–1362) [6,35] and is tightly regulated by Brd4 to enhance activation of RNA polymerase [36,37]. For in vitro Y102E binding assays, the CTM consisting of the last 138 amino acids of the Brd4 C-terminus (aa 1224–1362) was used. Since only the 20 amino acid Brd4 C-terminal peptide was used for co-crystallization, all possible contacts between E2 and the Brd4 CTM may not have been identified. Tyrosine phosphorylation was also detected at Y102 in the HPV-31 protein by mass spectrometry [38]. Unlike BPV-1, the phosphomimetic HPV-31 Y102E retained low level binding to E1 and stimulated transient replication. Similar to BPV-1 Y102E, HPV-31 Y102E did not bind the Brd4 CTM and maintain episomes [38].

**Figure 1:**
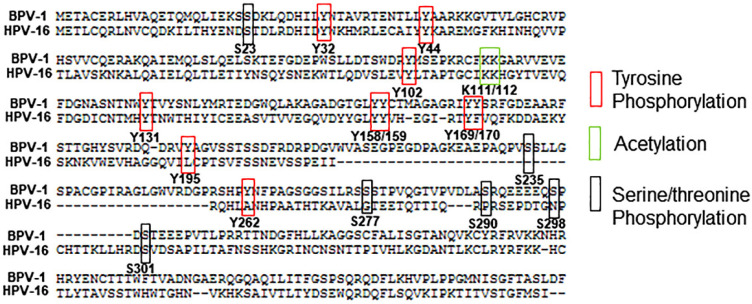
Summary of PTMs identified within BPV-1 E2 discussed in this review. Numbering of amino acids is based on BPV-1 E2 and was aligned to the HPV-16 E2 protein sequence using the Papilloma virus genome database (PaVE, pave.niaid.nih.gov) to show amino acid conservation [42].

The fibroblast growth factor receptors (FGFRs) were identified as binding partners to BPV-1, HPV-31, and HPV-16 E2 proteins in co-immunoprecipitation assays [11,12]. FGFRs are transmembrane tyrosine kinases and are activated by FGF ligands [39,40]. Although E2 Y102 was not a target of phosphorylation by FGFR2 or FGFR3, several other tyrosine residues were identified by mass spectrometry analysis of overexpressed BPV-1 E2 (Y32, Y44, Y131, Y158, Y159, Y169, Y170, Y195, Y262) and all except Y262 were located in the TAD [11] (Figure 1). Tyrosine 138 was also recognized as a target for FGFR3 in HPV-31 E2 [41].

Proline-rich tyrosine kinase 2 (Pyk2), also known as protein tyrosine kinase 2 beta (PTK2B), is a calcium-regulated non-receptor tyrosine kinase and belongs to the focal adhesion kinase (FAK) subfamily. Pyk2 was identified as a binding partner for E2 in a yeast two-hybrid screen and in a high-throughput Gaussia princeps luciferase-based Complementation Assay (HT-GPCA) [43]. Pyk2 phosphorylates E2 in the same TAD beta-sheet region as FGFR3 but at an adjacent tyrosine, Y131 [44].

Interestingly, when HPV-31 E2 Y131 and Y138 were individually mutated to glutamate, they shared similar phenotypes. Both phosphomimetic E2 mutant proteins were unable to stimulate both transient and stable replication [41,44,45]. E1 can bind to HPV-31 Y138E and Y131E, but this association is about 50% of WT [41,44]. Despite these observations, E1 is still recruited to the viral origin [41] so deficiencies in the formation of E1 and E2 complexes do not explain E2 Y131E and Y138E replication defects. HR HPV-E2 proteins bind Brd4 in several regions. The TAD of E2 binds the CTM whereas the DBD of E2 binds to the basic residue-enriched interaction domain (BID) of Brd4 [46]. N-terminal phosphorylation sites (NPS) within Brd4 regulate association of Brd4 to only high-risk but not low-risk E2 proteins [46]. HPV-16 and HPV-31 Y131E and Y138E binds the full-length Brd4 since these mutations do not affect the E2 DBD. However, HPV-16 and HPV-31 Y131E and Y138E do not bind to the Brd4 CTM [41,44,45]. These studies suggest that interaction with Brd4 CTM is critical for the initiation of HPV replication since E2 mutants that are unable to engage the Brd4 CTM fail to initiate genome replication. Protein expression signatures from the human protein atlas database reveal that Pyk2 and FGFR3 expression is highest in the basal cells of the epithelium [47,48]. In early stages of the viral lifecycle, the viral episomes are maintained at a low copy number until differentiation-dependent genome amplification. Pyk2 and FGFR3 may regulate HPV replication early after basal cell infection to limit viral replication and may prevent over-replication of episomes initially after infection.

Essential regulatory sites in proteins are often conserved through the evolution of a protein. Through the examination of PV E2 protein sequences, in Table 1 we depict which tyrosine residues are conserved and, therefore, likely to be essential for either structure, protein–protein contacts, or regulation of protein functions. The most conserved tyrosines are located within the E2 TAD, with highest conservation at Y131, Y138, and Y167. This conservation even extends past human PVs into BPV-1 and mouse papillomavirus E2 proteins. Within the DBD, the tyrosines are much less conserved, and two of the tyrosines in HPV-31 show a preference for phenylalanine within the entire HPV genera (Table 1). Several high and low risk HPV E2 proteins were compared to determine if there is any tyrosine preference. Amino acids 32, 44, 102, 315, and 319 all show a much greater tyrosine amino acid preference in high-risk than low-risk PVs. Only amino acid 168 shows a preference for tyrosine in low-risk, compared to high-risk, PVs. When the tyrosine was not conserved, it was changed to phenylalanine, except for Y32 (100% H in low risk), and Y102 (100% W in low risk). Amino acids 32, 44, 102, 168, 315, and 319 with a strong high/low risk preference along with the highly conserved Y131, Y138, and Y167 are most likely modulating E2 function by phosphorylation.

Several high and low risk HPV E2 proteins were compared to determine if certain tyrosines are conserved within a PV genotype. From the PaVE database [42], twelve high risk E2 protein sequences were compared (HPV species: 16, 18, 31, 33, 35, 39, 45, 51, 52, 56, 58, 59) to twenty-six low risk E2 proteins: (2, 6, 7, 11,13, 27, 28, 29, 32, 40, 44, 57, 61, 62, 72, 74, 77, 81, 83, 84, 86, 87, 89, 90, 91, 106) using ClustalW2 [49]. MM: Old harvest mouse, MMu: New laboratory mouse strain.

## 4. Acetylation

Acetylation involves the transfer of the acetyl group from acetyl coenzyme A onto lysine residues of the protein, which is catalyzed by lysine acetyltransferases or KATs. Acetylation and deacetylation of a protein is maintained by the activities of the KATs and lysine deacetylases (KDACs). KAT enzymes target a wide range of substrates from histones to viral proteins. Acetylation of proteins is a highly specific PTM that controls many cellular processes [50]. Studies have now been expanded to analyze acetylation on viral proteins and how these modifications can regulate the viral lifecycle.

BPV-1 E2 is acetylated by the lysine acetyltransferase p300 [51], which belongs to the histone acetyltransferase family of KAT proteins. Further mutational analysis found that p300 mediated acetylation of E2 occurs through a highly conserved lysine at residue 111 within the E2 TAD. Acetylation of K111 activates E2 dependent transcription [51]. Mutations to prevent acetylation (lysine to arginine) at residues 111 (K111R) and 112 (K112R) were partially mislocalized to the cytoplasm [51]. CRPV E2 mutated at K112 to Q, A, or R were able to localize to the nucleus, however, the K111 mutations to Q, A, or R were mislocalized to the cytoplasm. In combination with a nuclear localization signal (NLS), these K111 E2 mutants still could not activate transcription [52]. This suggests that lysine at 111 is important for E2 localization and these activities may be regulated by acetylation.

Unlike BPV and CRPV E2, K111 acetylation is not necessary for nuclear localization of HPV-31 E2 [53]. Mutation of HPV-31 E2 K111 to arginine was unable to stimulate transient and stable replication because unwinding of the viral origin was impaired [53,54]. Overexpression of the KAT p300 but not CREB binding protein (CBP) enhanced replication of HPV-31 WT E2 but not K111R suggesting that K111 is targeted by p300 to activate viral replication [54]. Using ChIP assays, HPV-31 E2 K111R was incapable of recruiting topoisomerase I to the ori to relieve double stranded DNA torsion during viral replication [54]. It has been reported that p300 levels are low in basal cells but high in the differentiated epithelium [55] and that p300 induces keratinocyte differentiation [56]. p300 acetylation of E2 may be necessary to allow robust genome amplification late in the viral lifecycle, but this has not been tested.

Several studies have demonstrated the importance of the class III histone deacetylase SIRT1 during the HPV lifecycle. SIRT1 protein expression is modulated by PV E6 and E7 [57,58]. In the episomal HPV-31 CIN612 cells, SIRT1 is necessary for genome maintenance and amplification by modulating histone acetylation and recruitment of homologous recombination repair factors NBS1 and Rad45 [57]. SIRT1 is in complex with HPV-16 E1 and E2, and represses HPV-16 replication [59]. Knockout of SIRT1 also increases acetylation of HPV-16 E2 [59]. Further studies are needed to determine if E2 is a direct target of SIRT1 deacetylase activity.

E8^E2 interacts with class I histone deacetylases (HDAC1, HDAC2, and HDAC3) in vitro [60]. The interaction is dependent on amino acids 3–12 of E8^E2, which do not overlap with any E2 amino acids. These HDACs contribute to transcriptional repression but there is no direct evidence of deacetylation of E8^E2 or E2 by these HDACs [60]. Future studies are needed to reveal the deacetylases responsible for regulating E2 acetylation.

## 5. Sumoylation

The post-translational modification SUMO (small ubiquitin-related modifier) occurs mostly on nuclear proteins and can regulate protein stability, localization, and function [61]. Sumoylation is a multi-step reversible process involving three classes of enzymes (E1-activating enzyme, E2-conjugating enzyme, and E3-sumo ligase). The enzyme Ubc9 transfers the SUMO substrate to a lysine residue. The SUMO modification alters the inter- or intramolecular interactions of the substrate resulting in dynamic changes in protein function. BPV-1, HPV-11, HPV-16, and HPV-18 E2 proteins interact with Ubc9 [62]. Further mapping revealed that mutation of lysine 292 to arginine within HPV-16 E2 lost sumoylation potential and this mutation is defective for transcription even though it maintains DNA binding activity and nuclear localization [62]. BPV-1 E2 K111 and K322 were found to be predicted SUMO substrate sites [62]. During keratinocyte differentiation, sumoylation activity is elevated [63] and it is thought that during genome amplification sumoylation of E2 protein may increase its stability and lead to increased replication [64].

## 6. Ubiquitination

The ubiquitin proteasome pathway is responsible for intracellular protein degradation in eukaryotic cells. It involves the addition of ubiquitin molecules to protein substrates at lysine residues to target protein degradation using a three-enzyme process. The process begins after activation of the E1 enzyme and sequential binding and transferring of the ubiquitin to an E2 ubiquitin-conjugating enzyme. The E2 enzyme binds to the E3 ubiquitin-protein ligase to associate to the target protein and transfer the ubiquitin to targeted lysine residues [65,66].

It has been shown that HPV-16 E2 interacts with the MDM2 E3 ligase through amino acids 322–335 in the DBD. Interestingly, this interaction does not increase E2 degradation but enhances its transcriptional activator function. Instead, E2 is degraded by the ubiquitin-proteasome pathway through ubiquitination of its TAD [67]. Pulse-chase experiments have revealed that the half-life of the BPV-1 E2 is 40 min [68] and 1 hr for HPV-18 E2 [68]. At the end of the G1 phase, E2 is degraded by the Skp1/Cullin1/F-box Skp2 (SCF(Skp2)) ubiquitin ligase by binding to the TAD of E2. The lysine residue(s) targeted by these enzymes are not yet known but it is speculated that phosphorylation of E2 is a prerequisite for SCF(Skp2) mediated degradation [69].

## 7. Future Perspectives

**Figure 2:**
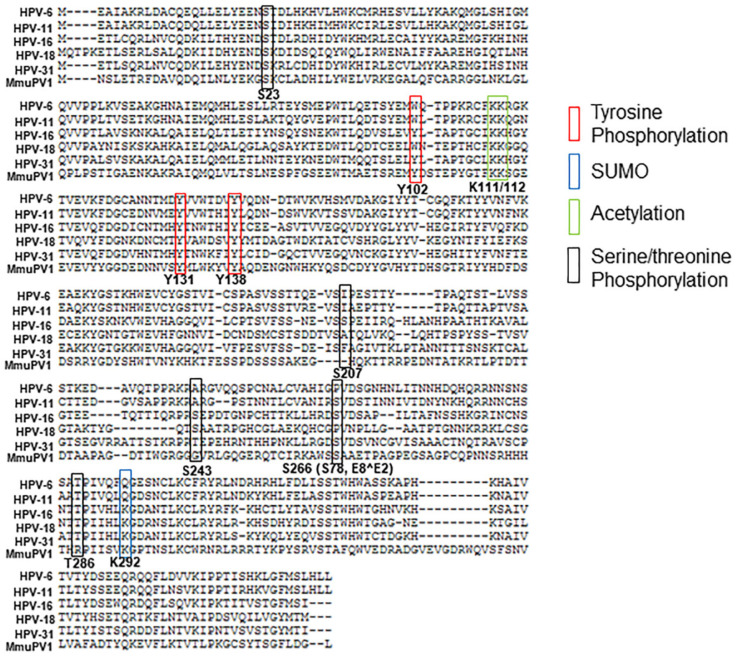
Summary of the PTMs identified within HPV-31 and HPV-16 E2 discussed in this review. Numbering of amino acids is based on the HPV-31 E2 sequence and aligned to HPV-6, HPV-11, HPV-16, HPV-18, and MmuPV1 E2 sequence from the PaVE database [42]. *S78 within E8^E2 is labeled as S266 in the HPV-31 E2 protein.

Sequence alignments of E2 proteins from genotypes using the PaVE [42] reveals conservation among possible PTM residues (Figure 2).

Unpublished mass spectrometry data from our laboratory has identified phosphorylation at conserved residues T/S81, T/S85, and Y344 in HPV-31 E2, and S23 in BPV-1 E2. We also detected acetylation at HPV-31 K68 and K334 residues. Future experiments will determine the necessity of these PTMs during E2′s regulation of the HPV replicative lifecycle.

Site-directed mutagenesis to change residues into PTM mimetics or unmodifiable forms has been very useful in understanding how E2 is regulated by these modifications. Most of these residues are also conserved in the mouse PV (MmuPV1) E2 protein (Figure 2 and Table 1). The MmuPV1 papillomavirus infection and tumorigenesis model presents a tractable model for studying these PTMs in vivo [70,71]. These experiments will provide critical insight to the function of the E2 PTMs during the viral lifecycle in a translational mouse model.

## 8. Conclusions

HPV carries out the distinct modes of replication in different layers of stratified epithelium. In the basal layers, viral genome replication is synchronized with the host cell cycle. In the non-dividing differentiated epithelium, the genomes undergo unrestrained amplification and generate infectious virions. One hypothesis is that E2 protein is elevated in differentiated cells, allowing robust E2 activity and replication during differentiation-dependent amplification. This hypothesis has only been tested for BPV-1 E2 in bovine warts and for HPV-16 in cervical lesions [19,72]. Recent data from our group and others suggest that regulation of E2 by PTMs are the molecular switches that control viral replication among the different phases of the viral lifecycle. Based on current understanding, phosphorylation and acetylation of E2 play pivotal roles in attaining and sustaining maintenance mode of genome synthesis. Acetylation of E2 by p300 at K111 activates replication through recruitment of Topo1 to the viral origin. Tyrosine phosphorylation within the TAD may restrict unlicensed replication through E2′s association with the Brd4 CTM. Serine/threonine phosphorylation in the E2 hinge region affects E2 stability rather than influencing direct replication mechanisms. However, several conserved serine/threonine residues are possible PTM targets within the TAD and DBD of E2, but their effect on HPV replication have not yet been fully studied.

Nearly all the experiments conducted involve over-expression of E2 to examine PTMs, which has implicit limitations. HPV initially infects the basal layer, and as the infection progresses, the cells differentiate into the spinous and granulosa layers. Each cell type supports different viral processes, and likely E2 is regulated by differing PTMs at each layer due to varying concentrations and activities of modifying enzymes. As an example, basal cells express low levels of tyrosine phosphorylated proteins [73] but high levels of phospho-serine peptides [74]. Upon differentiation, the levels switch and phospho-tyrosine increases [73] while phospho-serine decreases [74]. These changes may control E2 function through specific PTMs.

Many advances have been made in the last few years to study PTMs and to identify these specifically within E2. These include the development of phosphorylation and acetylation specific antibodies and the enhanced PTM detection/enrichment capabilities of mass spectrometry. Despite these advances, the lack of specific E2 antibodies to detect PTMs make it difficult to monitor these changes in vivo. Studying direct effects of PTMs on specific amino acids within E2 is limited to using mutagenesis to mimic or impair the PTM. Although useful, this is not an ideal system since it may not reflect the true PTM dynamics or the induced conformational changes within E2. Future experimental designs will need to include PTM specific antibodies to monitor E2 PTM turnover during the stages of the viral lifecycle.

## Figures and Tables

**Table 1:** Conservation of tyrosine residues in HPV.

HPV-31	12 High	26 Low	BPV-1	Mm/Mmu
Y19	92%Y 8%F	100%Y	Y19	Y/Y
Y32	75%Y	100%H	Y32	H/Y
Y44	75%Y 25%F	38%Y 46%F	Y44	Y/F
Y87	92%Y 8%F	77%Y	F87	Y/F
Y102	25%Y	100%W	Y102	W/Y
F121	33%Y 66%F	39%Y 62%F	Y121	F/Y
Y131	100%Y	96%Y	Y131	Y/Y
Y138	100%Y	85%Y 4%F	Y138	Y/Y
Y158	100%Y	85%Y 8%F	Y158	Y/V
Y159	100%Y	92%Y 4%F	Y159	Y/Y
Y167	92%Y 8%F	65%Y 15%F	Y169	Y/Y
F168	58%Y 42%F	100%Y	Y170	Y/Y
Y178	92%Y 8%F	100%Y	F180	Y/Y
Y310	100%Y	96%Y	F343	F/N
Y315	50%Y 8%F	15%Y	H349	H/T
Y319	75%Y 25%F	30%Y 70%F	Y353	Y/Y
Y344	83%Y 17%F	92%Y	F380	F/F
Y369	50%Y 17%F	46%Y 19%F	T405	S/T

## Data Availability

Not applicable.
